# Molecular Characterization of a New Wheat-*Thinopyrum intermedium* Translocation Line with Resistance to Powdery Mildew and Stripe Rust

**DOI:** 10.3390/ijms16012162

**Published:** 2015-01-20

**Authors:** Haixian Zhan, Xiaojun Zhang, Guangrong Li, Zhihui Pan, Jin Hu, Xin Li, Linyi Qiao, Juqing Jia, Huijuan Guo, Zhijian Chang, Zujun Yang

**Affiliations:** 1 School of Life Science and Technology, University of Electronic Science and Technology of China, Chengdu 610054, China; E-Mails: zhan030006@126.com (H.Z.); ligr28@uestc.edu.cn (G.L.); pzh1990@aliyun.com (Z.P.); 2 Crop Science Institute, Shanxi Academy of Agricultural Sciences, Taiyuan 030031, China; E-Mails: zxjemail@163.com (X.Z.); hujing56@126.com (J.H.); leexinlee@aliyun.com (X.L.); qiaoly1988@126.com (L.Q.); zhanhaixian@aliyun.com (H.G.); wrczj@126.com (Z.C.); 3 College of agronomy, Shanxi Agricultural University, Taigu 030801, China; E-Mail: jiajuqing@126.com; 4 Key Lab of Crop Gene Resources and Germplasm Enhancement on Loess Plateau, Ministry of Agriculture, Taiyuan 030031, China

**Keywords:** wheat, *Thinopyrum intermedium*, translocation line, powdery mildew, stripe rust, *in situ* hybridization

## Abstract

A new wheat-*Thinopyrum* translocation line CH13-21 was selected from the progenies derived from a cross between wheat-*Th. intermedium* partial amphiploid TAI7047 and wheat line Mianyang11. CH13-21 was characterized by using genomic *in situ* hybridization (GISH), multicolor-GISH (mc-GISH), multicolor-fluorescence *in situ* hybridization (mc-FISH) and chromosome-specific molecular markers. When inoculated with stripe rust and powdery mildew isolates, CH13-21 displayed novel resistance to powdery mildew and stripe rust which inherited from its *Thinopyrum* parent. The chromosomal counting analyses indicated that CH13-21 has 42 chromosomes, with normal bivalent pairing at metaphase I of meiosis. GISH probed by *Th. intermedium* genomic DNA showed that CH13-21 contained a pair of wheat-*Th. intermedium* translocated chromosomes. Sequential mc-FISH analyses probed by pSc119.2 and pAs1 clearly revealed that chromosome arm 6BS of CH13-21 was replaced by *Thinopyrum* chromatin in the translocation chromosome. The molecular markers analysis further confirmed that the introduced *Th. intermedium* chromatin in CH13-21 belonged to the long arm of homoeologous group 6 chromosome. Therefore, CH13-21 was a new T6BS.6Ai#1L compensating Robertsonian translocation line. It concludes that CH13-21 is a new genetic resource for wheat breeding programs providing novel variation for disease resistances.

## 1. Introduction

Powdery mildew caused by *Blumeria graminis* f. sp. *tritici* and stripe rust caused by *Puccinia striiformis* f. sp. *tritici* are devastating diseases of wheat growing in cold and wet regions of the world [1,2]. Although numerous genes conferring resistance to powdery mildew and stripe rust have been reported in wheat and its wild relatives, major resistance genes are rapidly overcome due to rapid virulence changes in the pathogen populations [3]. Therefore, it is essential for wheat breeding to continuously explore new genetic resources and transfer new genes from wild relatives that have potential resistance to powdery mildew and stripe rust.

*Thinopyrum intermedium* (Host) Barkworth and Dewey (genomes, JJ^s^St, 2n = 6x = 42), is a hexaploid species that confers potentially favorable traits that can be exploited for wheat genetic improvement [4]. *Th. intermedium* has wide range of adaptation to soils and climate and it displays a vast genetic diversity in the species [5]. The diversified *Th. intermedium* germplasms exhibited superior resistance to multiple wheat diseases including powdery mildew and stripe rust pathogens. *Th. intermedium* can be easily hybridized with common wheat, and a number of wheat-*Th. intermedium* derivatives were developed by chromosome engineering and breeding practices [6]. Previously developed wheat-*Th. intermedium* partial amphiploids including Zhong 1 to Zhong 5 [7,8], Otrastsjuskaya [9], TE-3 [10], TAI8335 [11] and TE253 [12] were reported to possess outstanding resistance to powdery mildew and/or stripe rust. However, it has not been reported so far that any wheat-*Th. intermedium* translocation lines carry both powdery mildew and stripe rust resistance genes.

A wheat-*Th. intermedium* partial amphiploid TAI7047 is effective against all existing Chinese powdery mildew and stripe rust races [11]. With the aim to transfer novel resistance traits to common wheat, TAI7047 was crossed with cultivar Mianyang11 (MY11) and the hybrids were backcrossed by using MY11 as recurrent parent. Among the obtained wheat-*Th. intermedium* derived progeniess, a new wheat-*Th. intermedium* introgression line CH13-21 showing high level of resistance to both powdery mildew and stripe rust was selected. In the present study, we determined the chromosomal constitution of the CH13-21 using multiple molecular cytogenetic approaches including the genomic and fluorescence *in situ* hybridization in combination with functional marker analysis.

## 2. Results

### 2.1. Morphology and Cytological Observations on CH13-21

Wheat-*Th. intermedium* derivative line CH13-21 was selected from the BC1F6 progenies of the crosses between wheat-*Th. intermedium* partial amphiploid TAI7047 and MY11. CH13-21 displayed similar agronomic traits to its wheat parent MY11. The height of CH13-21 adult plant was 75–80 cm, while TAI7047 was 100–110 cm. The CH13-21 produced 5–7 spikes per plant, and had higher tilling ability than the recipient parent MY11 (3–5 spikes), but lower than the donor TAI7047 (8–10 spikes). The root-tip chromosome counts and meiotic observation in pollen mother cells (PMCs) were made on 25 plants. The results showed that all CH13-21 plants had 42 somatic chromosomes and 21 bivalents at meiotic metaphase I of PMCs.

### 2.2. Powdery Mildew and Stripe Rust Resistance Testing

**Figure 1:**
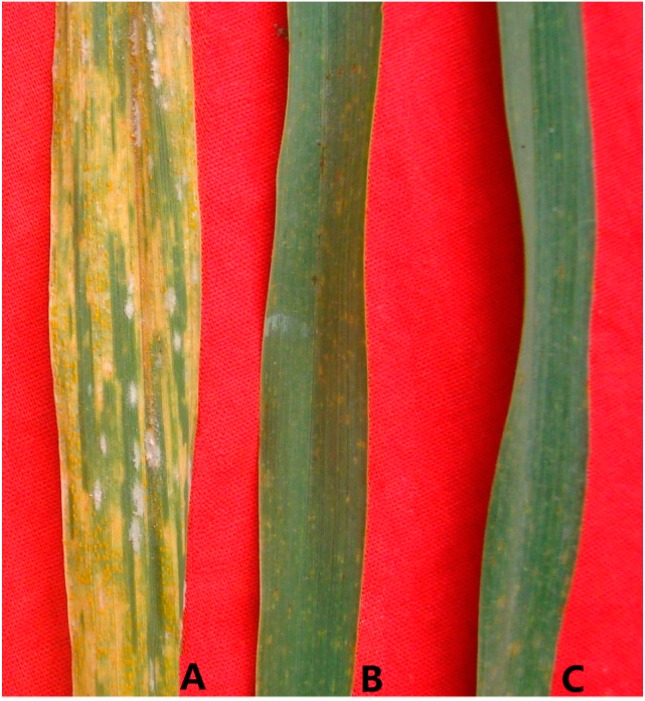
Response to stripe rust and powdery mildew of lines MY11 (**A**); CH13-21(**B**) and TAI7047 (**C**) on the leaves of adult plants.

Resistance testing data of infection types (ITs) at seedling stage demonstrated that CH13-21, TAI7047 and *Th. intermedium* were highly resistant to *B. graminis* f. sp. *tritici* (*Bgt*) races E09, E20, E21 and E26, whereas the wheat parent MY11 was highly susceptible (Table 1). At adult stages, all plants were inoculated with *Bgt* isolate and *P. striiformis* f. sp. *tritici* (*Pst*) races CYR30, CYR32, and CYR33. The field disease severity based on average relative area under the disease progress curve (AUDPC) were recorded among CH13-21 and its parents in comparison with the susceptible control Mingxian 169. A highly significant correlation (*r* = 0.926, *p* < 0.01) existed among the disease AUDPC readings at three different trials. TAI7047 and CH13-21 were resistance to all of these isolates (relative AUDPC < 1%), whereas the wheat parent MY11 was highly susceptible to both powdery mildew and stripe rust isolates (relative AUDPC > 90%). As shown in Figure 1, the similarity of final adult-plant resistance to two diseases were observed between TAI7047 and CH13-21. The results suggested the possibility that CH13-21 had retained the *Th. intermedium* chromatin carrying the resistance genes.

**Table 1:** Powdery mildew reaction on seedlings of CH13-21 and its parents.

Lines	Chromosome Number	Genome	Infection Types for *Bgt* ** to B isolates			
			E09	E20	E21	E26
*Th. intermedium*	42	StJJ^s^	0	0	0	0
TAI7047	56	ABD + (J + J^s^ + St) * J + J^s^ + St	0	0	0	0
Mianyang11	42	ABD	4	4	4	3
CH13-21	42	ABD	0	0	0	0

* indicated the mixed genome with chromosomes from three different genomic sets; ** indicated the infection types based on a 0–4 scale. Scores of 0–2 were classified as resistant and 3–4 as susceptible.

### 2.3. Genomic in Situ Hybridization (GISH)

**Figure 2:**
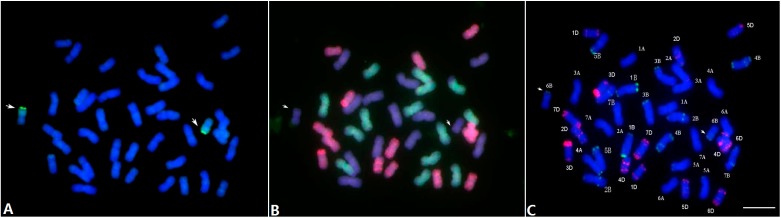
Genomic *in situ* hybridization (GISH) and sequential fluorescence *in situ* hybridization (FISH) patterns on the mitotic metaphase chromosomes of the common wheat line CH13-21. (**A**) GISH pattern with the labeled *Th. intermedium* genomic DNA as probe (green); (**B**) The multi-color (mc)-GISH pattern with labeled D-genomic DNA (red) and A-genomic DNA (green) as probes; (**C**) The mc-FISH pattern with the labeled pAs1 (red) and pSc119.2 (green) as probes. Arrows indicated the two translocated chromosomes, and scale bar showed 10 µm.

Genomic *in situ* hybridization (GISH) using the alien genomic DNA as probe provides an easy way to determine the origin of alien chromatin in the wheat background [13]. GISH analysis was performed on somatic chromosomes of CH13-21 by using genomic DNA of *Th. intermedium* as the probe and the Chinese Spring genomic DNA as the block (Figure 2A). The hybridized signals indicated that one pair of chromosomes consisted of long arms from *Th. intermedium* chromatin and short arms from wheat chromatin, suggesting that CH13-21 carried a pair of wheat-*Th. intermedium* translocated chromosomes. The translocation chromosome was occurred at the centromeric region and therefore the pair of chromosomes might be Robertsonian translocated chromosomes.

Multicolor-genomic *in situ* hybridization (mc-GISH) technique probed by D-genomic DNA and A-genomic DNA with B-genomic DNA as blocking, it can distinguish the A-, B- and D-genome chromosomes of wheat at the same time [8]. As shown in Figure 2B, mc-GISH displayed by A genome as green, D genome as red while the B genome as blue, respectively. Apparently, the result indicated that line CH13-21 contained complete 14 chromosomes of each A, B and D genomes. Compared with the GISH result in Figure 2A, we found that the translocated segments in CH13-21 were involved in a pair of B-genome chromosomes.

### 2.4. Fluorescence in Situ Hybridization (FISH)

The multi-color FISH (mc-FISH) using the high repetitive sequences as probes can not only effectively identify individual chromosomes of wheat, but also precisely determine chromosome elimination and addition in wheat-alien introgression lines [14]. The mc-FISH analysis with probes pAs1, detecting wheat D-genome chromosomes, and pSc119.2, mainly identifying B-genome chromosomes, were used to detect the chromosome constitution of CH13-21 (Figure 2C). The results clearly showed that a pair of long arm on chromosome 6B were replaced by the *Th. intermedium* chromatin. Based on the GISH and sequential mc-FISH results, we thus concluded that the CH13-21 was a wheat-*Th. intermedium* translocation line involving translocation between *Th. intermedium* chromatin and wheat chromosome 6B.

### 2.5. Molecular Marker Analysis

**Figure 3:**
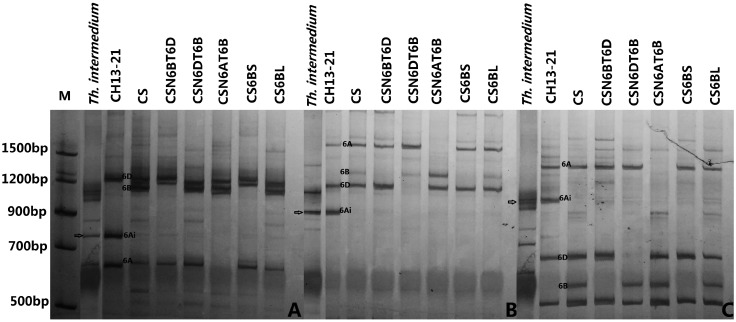
PCR amplification using primers TNAC1726 (**A**); TNAC1702 (**B**) and TNAC1752 (**C**). The arrows indicates the *Th. intermedium* specific bands.

The PCR-based landmark unique gene (PLUG) primers can amplify the diagnostic fragments in wheat homoeologous groups of individual chromosomes, and further determine the presence and absent of the specific chromosomal segments [15]. A total of 155 PLUG markers distributed over wheat homologous groups 1-7 were used to amplify the polymorphic bands among CH13-21, MY11, TAI7047 and *Th. intermedium*. We found that 10 PLUG markers from wheat homoeologous group 6 generated *Th. intermedium* specific bands in CH13-21, compared to those of its parents MY11 and TAI7047 (Table 2). As shown in Figure 3, the PLUG primers TNAC1752 and TNAC1702 amplified specific fragments from chromosomes 6A, 6B, and 6D of wheat revealed by Chinese Spring (CS) nulli-tetrasomic lines. The amplification demonstrated that the 6BL specific fragment was absent in CH13-21, which is consistent with the result of chromatin 6BL being absent in line CH13-21 revealed by FISH (Figure 2). The amplification also indicated that *Th. intermedium*-specific bands appeared in line CH13-21, suggesting that the *Th. intermedium* chromatin in CH13-21 attributed to homoeologous group 6 (Table 2, Figure 3). Therefore, we concluded that the CH13-21 was a wheat-*Th. intermedium* T6BS.6Ai#1L translocation line.

**Table 2:** PCR-based landmark unique gene (PLUG) markers for amplification of CH13-21.

Primer Name	EST	Primer Sequence (5'–3')	Wheat Bin Map	Enzyme Used	CH12-21 Specific Band
TNAC1748	BQ170604	F: TCGTAGAATTGGTCGACGATG R: ATGGATTGGCAAAGAAAGATG	6AL7-0.88-0.90	*Taq*I	550 bp
			6BL8-0.66-0.70		
			6DL1-0.47-0.68		
TNAC1763	CK197357	F: CGATTGGCCGTACAACTTTC R: TTGATGACGTTGAAGGGTCTC	6AL8-0.90-1.00	*Taq*I	900 bp 1100 bp
			6BL1-0.70-1.00		
			6DL10-0.80-1.00		
TNAC1768	CV765384	F: CCAGACGATACTGTGCATTCC R: GGTGTCCAGCCAGAGTTTATG	6AL8-0.90-1.00	*Hae*III	850 bp
			6BL1-0.70-1.00		
			6DL10-0.80-1.00		
TNAC1741	BE430390	F: GTCCCTGTTGGCTGTCTT R: CTTGAGTGTCGCATCGGAAG	6AL7-0.88-0.90	*Hpa*II	1200 bp 950 bp
			6BL8-0.66-0.70		
			6DL1-0.47-0.68		
TNAC1711	BU672205	F: CTTAAATCGCCTTTCCCACTC R: GACATTGCAAGGAGGAACAAG	C-6AL4-0.55	*Hae*III	1180 bp *
			C-6BL3-0.36		
			6DL6-0.29-0.47		
TNAC1702	CK157364	F: CATGGAAAGGTTGACAAGGAA R: CTGGATGTTCCATTTCTGCTC	C-6AL4-0.55	*Taq*I	750 bp *
			C-6BL3-0.36		
			6DL6-0.29-0.47		
TNAC1726	CK206456	F: CTCAACATCCACGAGTACCAG R: TTTGAAAGTTCCCAATCCAC	C-6AL4-0.55	*Taq*I	650 bp *
			6BL3-0.36-0.40		
			6DL6-0.29-0.47		
TNAC1751	DR73878	F: CTTCCTTTGCTTGTGATCCTG R: GCCTGAGGACTTGAAGTGGTA	6AL8-0.90-1.00	*Taq*I	800 bp
			6BL1-0.70-1.00		
			6DL12-0.68-0.74		
TNAC1740	CK206466	F: CGGAAGTGCTCGATTGTATCT R: GCGGGTTTCTTCTCAACCTT	6AL7-0.88-0.90	*Hpa*II	880 bp *
			6BL5-0.40-0.66		
			6DL6-0.29-0.47		
TNAC1752	CK158828	F: GTAGACGATGTCGAGGAGCAT R: CTTCACCAATTTCTCCCATGA	6AL8-0.90-1.00	*Taq*I	850 bp
			6BL1-0.70-1.00		
			6DL11-0.74-0.80		

* indicated the length is equal to 6B specific band.

## 3. Discussion

The production of wheat-*Thinopyrum* compensating Robertsoanian translocations with the targeted alien chromosomes by the centric breakage-fusion mechanism, is the first important step for transferring genes from *Thinopyrum* species to wheat for breeding purpose. Friebe *et al.* [16] firstly characterized radiation-induced wheat-*Th. ponticum* chromosome translocation lines and located the stem rust resistance gene *Sr26* on the translocation chromosome T6AS.6AL-6Ae#1L. Whelan and Hart [17] identified two independent Robertsonian translocations, involving the fusion between the short arm of a group 6 chromosome of *Th. Ponticum* and the long arm of chromosome 6D by using chromosome painting and PCR analysis. A gene for resistance to wheat streak mosaic *Cmc2* was located on the compensating translocated chromosomes T6DL·6Ae#2S [17] and T6AL·6Ae#2S [18]. Hu *et al.* [19] reported that a wheat-*Th. ponticum* 6J^s^(6B) substitution line X005 carried novel stripe rust resistance gene(s) from chromosome 6J^s^. Recently, Tang *et al.* [20] reported that a powdery mildew resistance line 08-723 possessed a fusion of homozygous translocation between St-chromosomes of *Th. intermedium* and chromosome 6A of wheat. Therefore, the *Thinopyrum* homologous group 6 chromosomes contained novel disease resistance genes and easily compensated the wheat homologous chromosomes. In the present study, the CH13-21 contained *Th. intermedium* chromatin 6Ai#1L carrying both powdery mildew and stripe rust genes by spontaneous chromosome translocation which originated from the crosses between wheat and wheat-*Th. intermedium* partial amphiploid. It is interesting to further identify new resistance genes in homologous group 6 chromosomes of *Thinopyrum* genomes.

Recently described wheat-*Th. intermedium* cryptic translocations carried two powdery mildew resistance genes, *Pm40* on 7B [21] and *Pm43* on 2D [22], as well as two stripe rust resistance gene, *Yr50* on 4B [23], and *YrL693* on 1B [24]. They were presumed to be transferred from *Th. intermedium* to common wheat on cryptic translocations. There is no report on powdery mildew or stripe rust resistance genes that have been transferred from a group 6 of *Th. intermedium* chromosome to wheat. The CH13-21 displayed novel resistance to multiply powdery mildew and stripe rust races at both seedling and adult plant stages. It will be interesting to reveal the novel genes or gene complex conferring the Pm and/or Yr resistance on chromosome 6Ai#1L in CH13-21. Moreover, pyramiding multiple and effective resistance genes is a potential method for durable resistance of genetic control [25]. The alien resistance genes complex such as the *S. cereale* 1RS-derived *Pm8/Yr9/Lr26* and the *Ae. ventricosa* 2N^v^ derived *Yr17/Lr34* confirmed to have multi-diseases resistance, which are effective to long-term resistance for several decades of global wheat breeding [2,16]. It is worthwhile to further investigate the wheat-*Th. intermedium* translocation line CH13-21 for possibly long-lasting resistance to wheat against stripe rust and powdery mildew.

Characterization of alien chromatins in wheat background is essential for effectively utilized new germplasm in breeding programs. The C-banding, GISH and the sequential FISH have been used widely for rapid identification of alien chromatins in wheat [14,26,27]. In the present study, the GISH and FISH were used to visualize the chromosome constitution of the wheat-*Th. intermedium* translocation line CH13-21 (Figure 2). However, The *in situ* hybridization was not able to precisely determine the homologous group of alien chromatin on wheat background. The PCR-based landmark unique gene (PLUG) primers are developed based on the orthologous gene conservation between rice and wheat, and presumably amplify fragments corresponding to the A, B and D genomes of wheat [15]. Our previous and other studies also confirmed that the PLUG markers have been used as effective tools for assigning the chromosomal homology of alien chromatins including *Dasypyrum*, *Secale* and *Thinopyrum* species [19,28,29,30]. Moreover, the PLUG primers were effective to detect the physical location of alien chromatin in wheat background [15]. In the present study, the PLUG markers were used to identify *Th. intermedium* alien fragments and revealed the alien chromosome arm belonging to homology group 6 in the wheat-*Th. intermedium* derivative line CH13-21. The molecular evidence confirmed that the translocation line CH13-21 conferred potential resistance genes which originated from its wild donor *Th. intermedium*. The PLUG markers specific to *Th. intermedium* chromosome 6Ai#1L, can be used as marker assisted selection for this Robertsonain translocation in the future wheat breeding. The CH13-21 can be used for further chromosome engineering by shortening the *Th. intermedium* segment in line CH13-21, using *ph1b* induced homoeologous recombination for wheat resistance breeding.

## 4. Experimental Section

### 4.1. Plant Materials

*Th. intermedium* accession PI440125 was provided by National Plant Germplasm System (NPGS) at Aberdeen, Idaho, USA. Wheat-*Th. intermedium* partial amphiploid TAI7047, wheat cultivars Mianyang11 (MY11) and Chinese Spring (CS) were maintained by Shanxi Academy of Agricultural Sciences (China). Wheat Chinese Spring nullisomic-tetrasomic lines (CSN6AT6B, CSN6BT6D and CSN6DT6B), and ditelosomic lines (CS6AS and CS6AL) were obtained from Dr. Bernd Friebe, Wheat Genetics and Genomics Resource Center, Kansas State University, USA.

### 4.2. Powdery Mildew and Stripe Rust Resistance Testing

Seeding of the wheat line CH13-21 and its parents were evaluated using four *B. graminis* f. sp. *tritici* (*Bgt*) isolates (E09, E20, E21 and E26). Inoculations were performed when the first leaves were fully expanded. The inoculated seedlings were kept in a dew chamber. Powdery mildew responses were described following 0–4 infection types scale with the highly susceptible cv. Mingxian 169 as a control [22]. Adult plants resistance were evaluated using *Bgt* races E09 and *P. striiformis* f. sp. *tritici* (*Pst*) strains CYR-30, CYR-32 and CYR-33 [23], respectively. The adult plant testing and the spreaders cv. Mingxian 169 were artificially inoculated three times at the seedling to tillering stages. Ten plants per row of 1.5 m were grown in the field trial with three duplication. The disease severity was first recorded when the susceptible control Mingxian 169 displayed 100% severity. Two additional disease readings were recorded at 10 day intervals. Based on the three disease severity, the calculation of the area under the disease progress curve (AUDPC) and the relative AUDPC for final adult-plant disease rating followed by the description of Ma *et al.* [31].

### 4.3. Genomic in Situ Hybridization (Gish) and Fluorescence in Situ Hybridization (FISH)

The chromosome spreads of materials were prepared through the methods described by Han *et al.* [32]. For GISH, the genomic DNA of *Th. intermedium* labeled with Alexa Fluor-488-5-dUTP (Vector Laboratories, Burlingame, CA, USA), and sheared genomic DNA of Chinese Spring wheat was used as blocking. For multi-color GISH (mc-GISH) to identify the A, B and D genome of wheat, the genomic DNA of *Triticum uratu* (A genome) and *Aegilops tauschii* (D genome) were labeled with Alexa Fluor-488-5-dUTP and Texas Red-5-dUTP (Vector Laboratories), respectively. The genomic DNA of *Ae. speltoids* (B genome) used as block. For FISH, the repetitive sequences pAs1 [33] were labeled with Texas Red-5-dUTP, The repetitive sequences pSc119.2 [34] was labeled with Alexa Fluor-488-5-dUTP (Roche Diagnostics, Indianapolis, IN, USA). The GISH and FISH Probe labeling and *in situ* hybridization were also operated according to Han *et al.* [32]. Images for GISH and FISH were taken using Olympus BX51 microscope equipped with DP70 camera.

### 4.4. Molecular Marker Analysis

Total genomic DNA was extracted according to the SDS protocol by Yang *et al.* [35]. The PCR-based landmark unique gene (PLUG) primers were employed [36]. The PCR amplification conditions were: after 5 min of denaturation at 95 °C, amplifications were programmed for 32 cycles, each consisting of 45 s at 95 °C, 45 s at 57 °C, and 10 min at 72 °C, then incubation 10 min at 72 °C. The 3 μL aliquot of each PCR amplification products after enzyme digestion was separated in 8% non-denaturing polyacrylamide gels and visualized by silver staining as described by Hu *et al.* [19].
